# Bacterial Stress Responses as Potential Targets in Overcoming Antibiotic Resistance

**DOI:** 10.3390/microorganisms10071385

**Published:** 2022-07-09

**Authors:** Jirapat Dawan, Juhee Ahn

**Affiliations:** 1 Department of Biomedical Science, Kangwon National University, Chuncheon 24341, Gangwon, Korea; jirapat@kangwon.ac.kr; 2 Institute of Bioscience and Biotechnology, Kangwon National University, Chuncheon 24341, Gangwon, Korea

**Keywords:** bacterial stress response, antibiotic resistance, antimicrobial adjuvant, stress adaptation, therapeutic strategy

## Abstract

Bacteria can be adapted to adverse and detrimental conditions that induce general and specific responses to DNA damage as well as acid, heat, cold, starvation, oxidative, envelope, and osmotic stresses. The stress-triggered regulatory systems are involved in bacterial survival processes, such as adaptation, physiological changes, virulence potential, and antibiotic resistance. Antibiotic susceptibility to several antibiotics is reduced due to the activation of stress responses in cellular physiology by the stimulation of resistance mechanisms, the promotion of a resistant lifestyle (biofilm or persistence), and/or the induction of resistance mutations. Hence, the activation of bacterial stress responses poses a serious threat to the efficacy and clinical success of antibiotic therapy. Bacterial stress responses can be potential targets for therapeutic alternatives to antibiotics. An understanding of the regulation of stress response in association with antibiotic resistance provides useful information for the discovery of novel antimicrobial adjuvants and the development of effective therapeutic strategies to control antibiotic resistance in bacteria. Therefore, this review discusses bacterial stress responses linked to antibiotic resistance in Gram-negative bacteria and also provides information on novel therapies targeting bacterial stress responses that have been identified as potential candidates for the effective control of Gram-negative antibiotic-resistant bacteria.

## 1. Introduction

**Figure 1:**
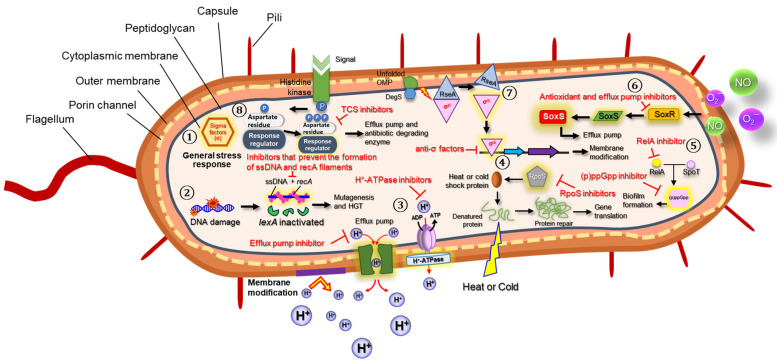
Overall scheme of bacterial stress responses in association with antibiotic resistance mechanisms. General stress response (①), SOS response (②), acid stress response (③), temperature stress response (④), starvation stress response (⑤), oxidative stress response (⑥), envelope stress response (⑦), and osmotic stress response (⑧). Single strand DNA (ssDNA), DNA repair-mediated gene (*recA*), horizontal gene transfer (HGT), adenosine diphosphate (ADP), adenosine triphosphate (ATP), stress response sigma factor (RpoS), homologue proteins (RelA and SpoT), guanosine pentaphosphate ((p)ppGpp), regulatory protein (SoxS), superoxide response regulon (SoxR), nitric oxide (NO), outer membrane protein (OMP), membrane anchored protease (DegS), anti-sigma factor (RseA), sigma factor E (σ^E^), and two-component systems (TCS).

Bacteria encounter a variety of adverse and harsh stresses in nature, such as nutrient limitation, osmotic pressure, extreme temperature, acid, and antimicrobials [1]. Bacteria are able to adapt to these unfavorable environmental stresses through protective mechanisms (Figure 1). The regulation of bacterial stress responses occurs at the transcriptional, translational, and post-translational levels, leading to changes in gene expression, protein activity, and cellular metabolism [1,2]. These bacterial stress responses mediate the resistance to stresses and the repair of cellular damage [2]. Gram-negative bacteria that are resistant to stresses possess various virulence factors, causing pneumonia as well as bloodstream and gastrointestinal infections [3]. The mechanisms underlying antibiotic resistance in Gram-negative bacteria include the reduction in membrane permeability, the alteration of target sites, the production of antibiotic-hydrolyzing enzymes, the increase in efflux pump activity, and the change in metabolic bypass [4]. In addition, multidrug resistance (MDR) is associated with transcriptional regulators involved in bacterial stress responses, for instance, *marA*, *soxS*, and *sdiA* [2]. The antibiotic resistance of *Enterobacteriaceae* is positively regulated by global transcriptional activators, including the Ara/XylS superfamily and the superoxide stress regulon [5,6].

Hence, stress response regulators can trigger the overexpression of efflux pump systems, such as the AcrAB system, that act as multidrug transporters for quorum-sensing signals and biofilm formation [7]. Nutrient limitation and amino acid starvation can lead to the evolution of resistance to polymyxin B and fluoroquinolones in Gram-negative bacteria [8,9]. Nutrient starvation can also induce biofilm formation, which leads to enhanced antibiotic resistance and is involved in chronic infections [10,11]. Other growth-limiting stresses, such as low pH and high temperature, induce several molecular rearrangements at the cellular metabolic level that are involved in the regulation of bacterial responses to antibiotics [12]. Bacterial stress responses not only encourage adaptation but also promote the virulence responsible for bacterial survival in stressful environments [13]. Therefore, bacterial stress responses may influence the development of antibiotic resistance in bacteria exposed to stressful conditions [2,14]. However, there is still a lack of information about the impact of bacterial stress responses on antibiotic resistance in bacteria. Presumably, bacterial stress responses can be potential targets for the control of antibiotic resistance. Therefore, this review discusses the role of bacterial stress responses in the development of antibiotic resistance in Gram-negative bacteria.

## 2. General Stress Response

**Figure 2:**
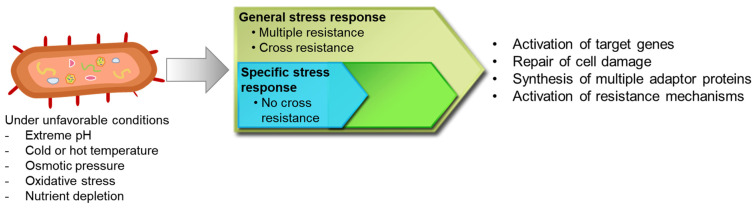
General and specific stress response regulation in bacteria.

**Figure 3:**
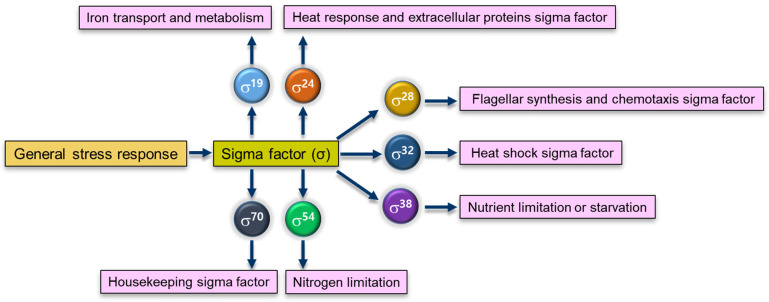
Sigma factors involved in the regulation of general stress responses.

Adaptive responses to stresses lead to the induction of specific gene expression as bacterial survival strategies. In addition, bacteria have general stress responses activated by transcriptional regulators under unspecific stress conditions (Figure 2) [1,15]. The general stress response can be mediated by several growth-limiting stresses, such as nutrient and oxygen deprivation, pH downshift, temperature changes, DNA damage, and high osmolality, involving more than 500 genes in Gram-negative bacteria [2]. The expression of stress-related genes is regulated by the RNA polymerase sigma factor (σ^s^) [16]. The σ^s^ is a key regulator for the general stress response in Gram-negative bacteria [17]. The σ^s^ encoded by *rpoS* is primarily responsible for the transcription of genes necessary for bacterial replication and growth [15]. Different alternative sigma factors play an important role in bacterial adaptation under stressful environmental conditions (Figure 3). These specific sigma factors direct RNA polymerase to recognize specific promoters of genes corresponding to environmental stresses [18].

The main role of the general stress response is to prevent and repair sub-lethal and lethal damage. In Gram-negative bacteria, the general stress response promotes survival under environmental stresses and also induces the expression of virulence factors [14,18]. For example, the σ^s^ acts like alternative sigma factors in Gram-negative bacteria, including *Burkholderia pseudomazei*, *Escherichia coli*, *Salmonella* Typhimurium, and *S. enteritidis*, to protect bacteria from different stress conditions and promote the expression of several genes required for cell survival in the stationary phase [18,19]. The pathogenicity of *S.* Typhimurium is associated with the σ^s^-dependent transcription of the *spv* gene cluster (Table 1) [18]. In addition, the expression of sigma factor 24, σ^24^, in *Pseudomonas aeruginosa* may modulate the production of the mucoid envelope, which defends against antibiotics, oxidative stress, and immunological assault [20]. Moreover, the extracytoplasmic function (ECF) sigma factor 70 (σ^70^) in *Neisseria gonorrhoeae* and *Caulobacter crescentus* may protect bacteria from oxidative damage through the expression of the gene that encodes MsrAB, which is responsible for methionine sulfoxide reductase activity [21]. The presence of the σ^s^ also affects the susceptibility of bacteria to antimicrobial agents and the induction of biofilm formation. The expression of the sigma-factor-related genes *pvdS* and *hasI* is responsible for iron acquisition and metabolism in *P. aeruginosa* and *E. coli* and can reduce susceptibility to various antibiotics, including carbapenems and vancomycin [22]. The resistance to carbapenems and vancomycin is due to the increased expression of the efflux pump, the decreased expression of porin, and the overproduction of carbapenemase enzymes [22]. Therefore, the expression of *pvdS* and *hasI* by ECF sigma factors results in the overexpression of the multidrug efflux system (MaxAB–OprM) and eventually leads to carbapenem and vancomycin resistance [22]. Furthermore, The ECF sigma factors also play a key role in regulating antibiotic resistance in *Klebsiella pneumoniae*, leading to increased resistance to cephalosporins and carbapenems [23].

Interestingly, sub-inhibitory concentrations of antibiotics, including aminoglycosides, β-lactams, and quinolones, induce σ^s^-dependent general stress responses and upregulate adaptive mutant genes in Gram-negative bacteria, resulting in the development of multidrug resistance [35]. Sub-inhibitory concentrations of β-lactams increase the cellular amount of the σ^s^ and induce RNA-polymerase-IV-dependent mutagenesis, which is responsible for the generation of mutagenic oxidized nucleotides under antibiotic treatment [35]. The σ^s^ also contributes to distinct changes in fitness cost of resistance. Mutation as a cause of antibiotic resistance is associated with fitness cost in Gram-negative bacteria due to the dysfunction of ribosome biogenesis [46]. Surprisingly, the σ^s^-mutant *S.* Typhimurium grows faster in a low-carbon medium than wild-type strains. The induction of the σ^s^ in wild-type bacterial cells retarded bacterial growth. However, the σ^s^-mutant was more susceptible to heat stress than the wild type, suggesting that the σ^s^ may contribute to long-term cell survival under growth-restricted conditions. On the other hand, a lower level of the σ^s^ in bacteria contributes to increased growth at low nutrient concentrations and enhanced susceptibility to external stress [46,47]. The fitness costs of resistance depend on growth conditions and genetic background [46]. Thus, understanding the stress-induced σ^s^ in association with fitness costs may help in developing strategies to control antibiotic resistance. Moreover, the σ^s^ is involved in flagellar synthesis, which is responsible for biofilm formation in the Gram-negative bacteria *Edwardsiella tarda* and *Yersinia pseudotuberculosis* [33]. As a result, the general stress response sigma factor, σ^s^, contributes to bacterial survival under stressful conditions and influences the expression of antibiotic resistance-related genes in Gram-negative bacteria (Table 1).

## 3. SOS-Response-Mediated Antibiotic Resistance

**Figure 4:**
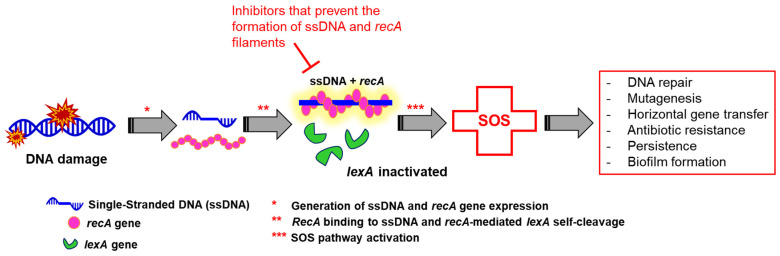
Bacterial SOS response pathway.

Environmental stresses, such as high pressure, acid, oxidants, nutrient limitation, and antibiotic exposure, can induce DNA damage indirectly through the formation of reactive oxygen species (ROSs) [48] or directly through interactions with DNA molecules [49]. The SOS response is a well-known bacterial stress response that is mainly induced by DNA damage [50]. The SOS response is initially triggered by an abnormal single-stranded DNA (ssDNA) that binds to RecA [51]. In fact, the SOS response is a DNA damage repair system that is associated with bacterial adaptability and pathogenicity (Figure 4) [52]. The ssDNA/RecA complex stimulates the proteolytic cleavage of the LexA repressor. LexA and RecA are SOS regulators that trigger the expression of genes encoding various DNA repair proteins [53]. In DNA-damaged bacterial cells, the accumulated *recA* single-stranded DNA (ssDNA)–ATP complex activates *lexA* for self-cleavage, which reduces the LexA protein in the bacterial cells and activates SOS gene expression (Figure 4) [53]. Accordingly, the *lexA* gene is a repressor, while the *recA* gene is an inducer [54,55]. The *recA* gene is also important for controlling swarming motility and bacterial behavior in biofilms and promoting homologous recombination [53]. Therefore, SOS responses play a key role in chronic bacterial infections by inducing biofilm formation and antibiotic resistance through mutagenesis and genomic rearrangement [52]. The SOS response upregulates integron integrases to increase cassette rearrangements carrying antibiotic resistance genes [56].

The damage in bacteria treated by fluoroquinolones induces the SOS response [57]. In addition, antibiotic pressure can increase the mutation rate in different pathways, including general stress, oxidative stress, and SOS responses [58]. Sub-inhibitory concentrations of β-lactams and fluoroquinolones have been shown to induce SOS responses and increase mutation rates in Gram-negative bacteria, including *E. coli*, *P. aeruginosa*, and *Vibrio cholera* [59,60]. The treatment of *E. coli* with sub-inhibitory concentrations of fluoroquinolones induces mutagenesis by stimulating ROS production, resulting in increased minimum inhibitory concentrations (MICs) for several antibiotics [61]. β-lactams can induce the SOS response via the two-component signal transduction system, *dpiBA*, to reduce antibiotic susceptibility by inhibiting cell division and increasing genetic variability [58]. In addition to mutations, horizontal gene transfer (HGT) can cause genetic variation in bacteria; this allows bacteria to acquire foreign DNA sequences from other species. In fact, conjugative transposons, also known as integrative conjugative elements (ICEs), have been found to be activated by the SOS response [62]. The ICEs efficiently transmit genes from donor cells to recipient cells; this phenomenon is responsible for antibiotic resistance, pathogenicity, and an alternate carbon metabolism [63]. Thus, HGT induced by SOS plays an important role in the distribution of genes that increase virulence and antibiotic resistance [64]. For example, the SOS response has been observed to activate HGT by activating the mobile genetic element SXT. SXT is an ICE-related element derived from *V. cholera* that confers resistance to several antibiotics, including chloramphenicol, streptomycin, sulphamethoxazole, and trimethoprim [65]. In addition, *bla*-encoding β-lactamases that hydrolyze β-lactam antibiotics can be transferred to antibiotic-sensitive bacteria through HGT, resulting in the emergence of new antibiotic-resistant bacteria [66]. Consequently, HGT plays an important role in the simultaneous transmission of numerous antibiotic resistance genes between species and within species [64]. The development of antibiotic resistance mediated by the SOS response may also be attributed to the induction of recombination events. The SOS response has been shown to induce the expression of an integrase that induces integrons, resulting in the activation of virulence genes encoding antibiotic resistance [62]. The SOS induction directly upregulates the expression of antibiotic resistance genes, such as *qnr*, which encodes quinolone resistance that directly targets DNA gyrase. The expression of these genes is regulated by the SOS response, and they possess a conserved *lexA* binding site in their promoters [67].

Another interesting connection between the SOS response and antibiotic resistance is the formation of biofilms and the induction of persistent cells. Antibiotic stress can induce the SOS response and then lead to biofilm formation [68]. The extracellular matrix of biofilms provides an antibiotic diffusion barrier so that bacteria are able to survive at a high concentration of antibiotics [60]. Additionally, up to 1% of bacterial cells in biofilms show a dormancy phenotype known as persistent cells that are highly resistant to antibiotics [52]. The SOS response is associated with the generation of persistent cells and an increase in antibiotic resistance in *E. coli*. The overexpression of SOS-inducible genes is associated with a decrease in antibiotic susceptibility. Moreover, the sub-MIC of SOS-activating antimicrobials can lead to resistance to unrelated antibiotics. In *P. aeruginosa*, ROSs and DNA-damaging agents induce biofilm formation in the SOS response [69]. The initial stage of biofilm formation is induced by the *lexA* regulon [70]. Furthermore, the activation of the SOS response increases the *recA* concentration in *Salmonella* spp., which subsequently impairs swarming motility [71].

The evolution of antibiotic resistance mediated by the SOS response requires the cleavage of the SOS repressor *lexA* and the modification of transcription and translation by *recA* in response to environmental stress. RecA is known as an SOS response inducer involved in HGT. Therefore, RecA is an ideal target for blocking SOS responses and can be used to design a therapeutic strategy to control antibiotic-resistant bacteria. Previous study has demonstrated that the deletion of *recA* showed a significant reduction in levofloxacin susceptibility and delayed the development of resistance in Gram-negative bacteria [72]. In addition, the combination of *recA* inhibitors and antibiotics enhanced antibiotic activity [73]. Moreover, small molecules that prevent the formation of ssDNA and *recA* filaments have been proposed as SOS inhibitors. For example, recent study has found that the use of zinc compounds could inhibit the *recA* regulon that binds to ssDNA, leading to the inhibition of the SOS response and the blockage of the transmission of β-lactam resistance genes [74]. Thus, the impact of *recA* on the SOS response and antibiotic susceptibility provides useful insights into a potential target in combating the emergence of antibiotic resistance. The induction of the bacterial SOS response can be inhibited by anti-SOS factors such as plasmid SOS inhibition (Psi) [51]. However, only a few previous in vitro screening efforts have been performed using *lexA* inhibitor compounds [75]. Hence, the lack of small molecules that inhibit *lexA* may be part of the challenge, which also includes the intramolecular nature of self-cleavage and the lack of understanding of the interface between *lexA* and *recA*.

## 4. Acid-Stress-Induced Antibiotic Resistance

**Figure 5:**
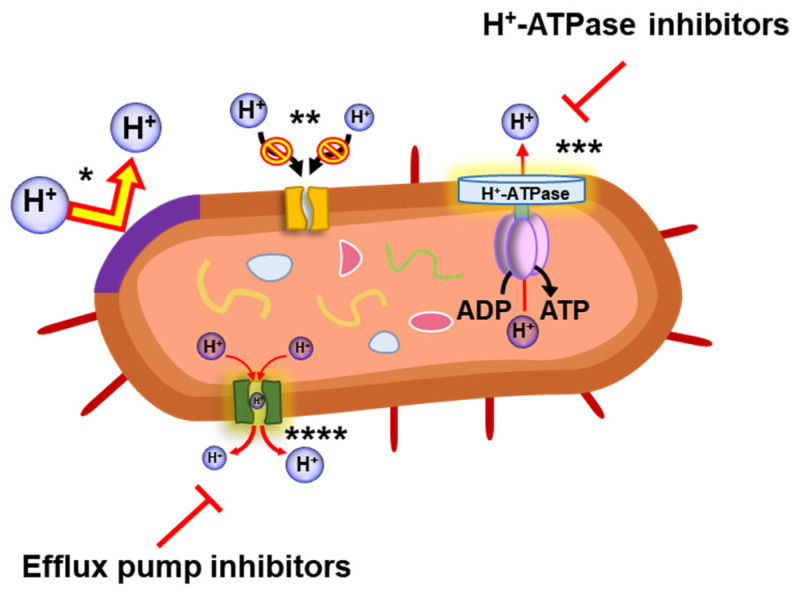
Bacterial acid stress responses, including the decrease in cell membrane fluidity (*), the modification of membrane channel size (**), the proton efflux by H^+^-ATPase mechanism (***), and the proton pump (****).

Acid stress is one of the most common environmental stresses, especially for Gram-negative *Enterobacteriaceae*, which are the normal inhabitants of the large and small gastrointestinal tracts [76]. The phenomenon of pH homeostasis is the regulation of intracellular and extracellular pH under acidic stress (Figure 5) [77,78]. Bacteria inhibit proton penetration by modifying the cytoplasmic membrane and regulating the size of membrane channels in an acidic environment [77]. Additionally, the expression of *gadBC* contributes to pH homeostasis, which can protect bacteria from acidic conditions [79]. Interestingly, the acid stress response is stimulated by antibiotics, leading to a decline in pH and the induction of RpoS [79]. The induction of the rapid acid stress response to trimethoprim protects bacteria exposed to acid by the expression of the GadBC operon responsible for maintaining intracellular pH [79]. Moreover, acid-tolerant bacteria have impermeable membranes to prevent proton influx into cells [80]. For example, *Acidithiobacillus ferrooxidans* increased the size of its outer membrane porins to maintain internal pH [81]. In addition, the proton-pumping ATPase (H^+^-ATPase) b is necessary for pumping protons out of bacterial cells [82]. The pH homeostasis mechanisms also play an important role in antibiotic resistance development due to involvement in antibiotic efflux and/or the alteration of antibiotic targets [83]. Bacteria utilize the proton-pumping system to transport antibiotic molecules out of the cell [84]. Additionally, the response to acid stress causes a change in membrane fluidity [80]. The bilayer structure is altered depending on the ratio of saturated and unsaturated fatty acids in the membrane of Gram-negative bacteria, resulting in the modulation of membrane fluidity [80,85]. A high percentage of unsaturated fatty acids in the bacterial membrane affects cell viability under acidic conditions [80]. Furthermore, the changes in membrane fluidity and lipid composition can protect bacteria by reducing the permeability of acids and antibiotics [86].

The bacterial efflux system induces cross-resistance to acids and antibiotics. Therefore, the inhibition of efflux pumps can be a potential target for the control of acid and antibiotic resistance in Gram-negative bacteria. For example, the efflux pump inhibitor promethazine (PMZ) induced a bacterial stress response to acidic pH by upregulating several genes [87]. The inhibition of efflux pumps in bacteria is associated not only with antibiotic susceptibility but also with the acid stress response [9,87,88,89]. Thus, it should be noted that the efflux pump inhibitor (EPI) acts as an adjuvant in combination with antibiotics. Furthermore, the EPI can disrupt the export of quorum-sensing molecules that modulate biofilm formation and limit the HGT of multidrug-resistant bacteria [90]. In addition, the use of H^+^-ATPase inhibitors, such as bicarbonate, transferrins, and *N*,*N*′-dicyclohexylcarbodimide, is able to enhance antibiotic activity against several Gram-negative bacteria [91,92]. The inhibition of ATPase activity and H^+^ translocation may cause the perturbation of intracellular pH, resulting in the modification of the proton gradient and leading to cell death [92]. Therefore, bacterial H^+^-ATPase may be a target in the development of new therapeutic strategies against antibiotic-resistant bacteria.

## 5. Heat- and Cold-Stress-Associated Antibiotic Resistance

**Figure 6:**
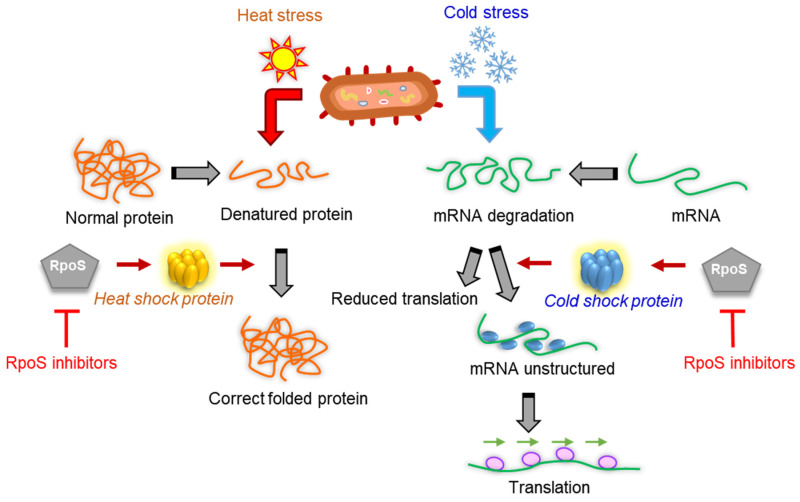
Heat and cold stress responses in bacteria.

Heat and cold are two of the most common environmental stresses for bacteria. The molecular responses of bacteria to sudden increases and decreases in temperature are called the heat shock response (HSR) and the cold shock response (CSR), respectively [93]. The HSR produces heat shock proteins (HSPs) when bacteria are exposed to heat (Figure 5) [94]. HSPs help restore the native structure of thermally unfolded proteins and induce proteasomal protein degradation [95]. The production of cold shock proteins (CSPs) is triggered by the response to a rapid temperature downshift (Figure 6) [48]. The CSP family consists of nine homologous proteins, including CspA through CspI [96]. The CSPs act as nucleic acid chaperones to promote bacterial translation initiation under cold stress [97]. A major HSP ClpL affects cell wall biosynthesis, leading to an increase in β-lactam resistance [98]. A protease ClpXP acts as a protein quality control system that is induced by heat shock and other stresses, which is associated with antibiotic resistance [99]. In addition, the HSR induces an increase in the rate of genetic recombination and HGT in class one integrons, which contribute to multidrug resistance in Gram-negative bacteria [100]. Therefore, heat stress can stimulate HGT, resulting in the acquisition of antibiotic resistance [101]. Furthermore, the CSPs improve bacterial translation at low temperatures, leading to the development of antibiotic resistance in bacteria [102]. For example, CspD contributed to the formation of biofilm and persister cells [103]. In addition, the alteration of porin expression blocked the entry of antibiotics into bacterial cells and developed an adaptive resistance to β-lactam antibiotics in *Moraxella catarrhalis* exposed to low temperature [104]. The expression of genes encoding membrane fusion proteins of the RND family of multidrug efflux pumps (*acrA* and *acrB*) was significantly increased in *M. catarrhalis* exposed to low temperature [105].

The heat- and cold-stress-induced HSPs and CSPs have gained attention as targets for controlling antibiotic-resistant bacteria. In fact, CSP- and HSP-disrupting agents have been developed to block the *N*-terminal ATP-binding pocket [106]. The DnaK belonging to the Hsp70 family contributes to bacterial multidrug resistance [107]. Therefore, DnaK inhibitors can effectively enhance antimicrobial activity and prevent antibiotic resistance. For example, the disruption of DnaK resulted in an increase in the susceptibility of *E. coli* to fluoroquinolones, oxacillin, and methicillin [108]. Consequently, the virtual screening strategy and the selection of ligands that bind to the relevant residues may be used to discover potential inhibitors that specifically degrade DnaK with high affinity. Moreover, the sigma factor *rpoS* is required for the induction of the o*tsA* and o*tsB* genes that upregulate CSPs through a CspA-independent pathway. In fact, the *rpoS* gene is responsible for the synthesis of CSPs and HSPs. Hence, the inhibition of *rpoS* can also be considered as an alternative target for the control of CSP and HSP synthesis and, by extension, antibiotic resistance.

## 6. Starvation-Stress-Associated Antibiotic Resistance

**Figure 7:**
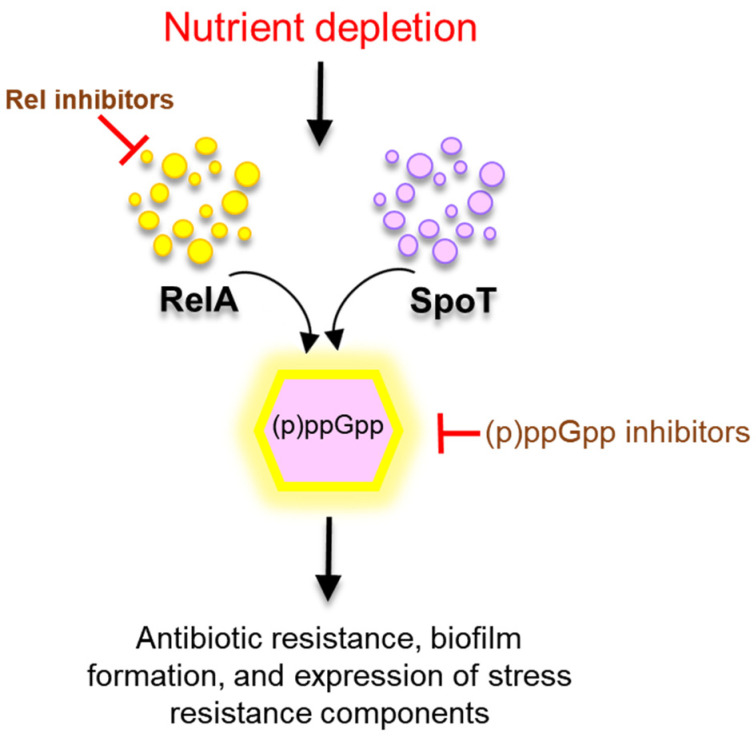
Bacterial nutritional stress response by the (p)ppGpp system.

Amino acid deficiency stimulates an adaptive response mechanism known as the stringent response (SR) [109]. The SR is mediated by (p)ppGpp, i.e., alarmones such as guanosine 50′-(tri)diphosphate and 30′-diphosphate, that act as messengers of amino acid starvation [110]. In the SR, these hyperphosphorylated guanosine derivatives are the key effector molecules synthesized or hydrolyzed by the RelA/SpoT homolog (RSH) superfamily (Figure 7) [111]. The alarmone (p)ppGpp plays a vital role in regulating the transcription of genes required for bacterial virulence, pathogenicity, and long-term survival [112]. Importantly, (p)ppGpp is also associated with antibiotic tolerance and biofilm formation in Gram-negative bacteria under nutrient deprivation [9]. In addition, (p)ppGpp plays an important role in β-lactam resistance. The roles of (p)ppGpp in the development of β-lactam resistance include the inhibition of peptidoglycan metabolism and the elimination of penicillin-binding proteins [110]. Other antibiotic-resistance-inducing mechanisms include endogenous mutations and the acquisition of foreign DNA [113]. Therefore, the deletion of the *relA* and *spoT* genes reduced mutation rates in *E. coli* and suppressed fluoroquinolone-resistant colonies in *P. aeruginosa* [4,9,114].

The activation of the SR leads to a reduction in metabolic processes and enhanced antibiotic resistance in Gram-negative bacteria. Therefore, (p)ppGpp has led to a growing interest in the development of SR inhibitors. Relacin, a 2′-deoxyguanosine-based analog of ppGpp, inhibits Rel-mediated (p)ppGpp synthesis, leading to bacterial cell death and the inhibition of biofilm formation [115]. Interestingly, vitamin C is a potential substrate for (p)ppGpp inhibitors and can competitively bind with Rel [116]. Recently, a high-throughput screening method was used to discover Rel inhibitors by using Rel from *Mycobacterium tuberculosis* and a new (p)ppGpp synthesis assay based on the quantification of adenosine 5′-monophosphate (AMP) [117]. Therefore, this approach can be used to design new anti-(p)ppGpp compounds that can control antibiotic-resistant bacteria. An alternative strategy for controlling the SR is to inhibit the accumulation of (p)ppGpp. The synthetic cationic *L*-amino acid peptide effectively degrades (p)ppGpp and prevents biofilm formation in Gram-negative bacteria, including *E. coli*, *P. aeruginosa*, *K. pneumoniae*, and *A. baumannii* [118]. The newly synthesized peptides can prevent the accumulation of (p)ppGpp and promote the degradation of (p)ppGpp by directly binding to (p)ppGpp [119].

## 7. Oxidative-Stress-Associated Antibiotic Resistance

**Figure 8:**
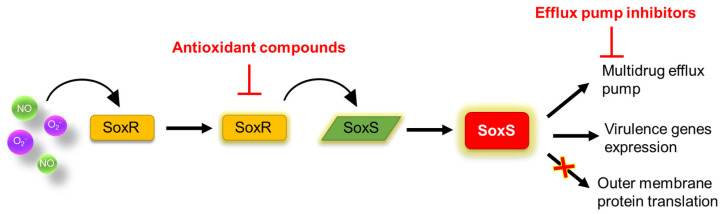
Bacterial oxidative stress response.

Adaptive oxidative stress responses are the survival strategies of bacteria when exposed to high levels of ROSs, such as peroxides, superoxide, the hydroxyl radical, and singlet oxygen [109,120]. Oxidative stress triggers the efflux pump systems that contribute to antimicrobial resistance [109]. The adaptive superoxide (SO) stress response is regulated by SoxRS that is activated by oxidized SoxR (Figure 8). The AcrAB–TolC multidrug efflux system plays a major role in the SoxRS-mediated response as a redox-responsive regulator. Indeed, the AcrAB–TolC system contributes to the response to redox-cycling agents mediated by SoxRS [121]. Not surprisingly, SoxS regulates the expression of acrAB in Gram-negative bacteria [121,122]. SoxS also regulates small interfering RNA (siRNA; *micF*) to repress the translation of outer membrane protein F (OmpF), leading to reduced antimicrobial uptake [121]. Moreover, the SoxRS-mediated response is also involved in the expression of genes encoding the core oligosaccharide of lipopolysaccharides (LPSs), resulting in enhanced resistance to β-lactams, fluoroquinolones, and macrolides [109]. Another multidrug efflux system regulator associated with oxidative stress is the global regulator MrgA, which regulates certain efflux transporters, namely *norA*, *norB*, *norC*, *norD*, *mdeA*, *lmrS*, and *sdrM*, belonging to the major facilitator superfamily (MFS) [123]. This implies that oxidative stress activates antibiotic resistance through an MgrA-mediated redox sensing pathway. Therefore, ROSs and low pH induce an MrgA-associated increase in multidrug efflux pumps, leading to enhanced antibiotic resistance [124]. In addition, it has been demonstrated that oxidative stress induces the RND family in Gram-negative bacteria [125]. The overexpression of MexXY genes induced by oxidative stress and ribosome-targeting antibiotics is associated with increased resistance to fluoroquinolones, β-lactams, macrolides, tetracycline, and aminoglycosides [126,127]. The CmeABC multidrug efflux pump is also induced by ROSs in *Campylobacter jejuni* [128]. Accordingly, the ROS-mediated efflux pump system can be a possible target in the effort to overcome antibiotic resistance in bacteria.

The suppression of efflux pumps, such as AcrAB–TolC system, increases the tolerance of oxidative stress and the susceptibility to antibiotics in bacteria. Therefore, efflux pump inhibitors (EPIs) are a very attractive target in the search for antimicrobial drugs to control antibiotic-resistant bacteria. Several efflux systems have been demonstrated to be inhibited by Phe-Arg β-naphthylamide (PAβN), and arylpiperazines have a strong inhibitory effect on RND efflux pumps [129]. Furthermore, a variety of antimicrobial discovery techniques were employed to find safe and effective inhibitors of the AcrAB–TolC system. For example, ligand docking studies were used to investigate the binding ability of ArcAB substrate and inhibitor compounds in different AcrAB crystal structures. According to the ligand study, diaminoquinoline acrylamide was demonstrated as a potent AcrAB–TolC inhibitor by binding to the membrane fusion protein AcrA and enhancing the antibacterial activities of novobiocin and erythromycin [130]. In addition, the well-known antiemetic drug domperidone is considered a promising ArcB inhibitor because it increases the antibiotic susceptibility of MDR *E. coli* strains to levofloxacin and ciprofloxacin [131]. Therefore, diaminoquinoline acrylamide and domperidone can be considered alternative EPIs and may be used as adjuvants with conventional antibiotics in order to control antibiotic-resistant bacteria. Another interesting observation was that the expression of the AcrA and SoxS genes was significantly reduced in the combination of honey, plant alkaloid extract, and ciprofloxacin compared to ciprofloxacin treatment alone [132]. It has been shown that plant alkaloid extracts have EPI activity, while the flavonoids and carotenoids in honey have antioxidant activity [133]. This fact may imply that the presence of these exogenous antioxidants in honey samples leads bacteria cells to reduce the expression of endogenous antioxidant genes, such as *soxS* [132]. Thus, the synergistic effect between honey and plant alkaloid extract can improve ciprofloxacin activity by inhibiting efflux pump AcrAB–TolC and reducing the oxidative stress response. This observation opens the way for the development of novel antibiotic combinations. Although there is a risk of impaired antibiotic efficiency due to an induced expression of efflux pumps, the combined use of antibiotics with compounds that disturb cellular redox homeostasis could nevertheless significantly enhance antibiotic activity.

## 8. Envelope-Stress-Mediated Antibiotic Resistance

The Gram-negative bacterial envelope, consisting of the cytoplasmic membrane (symmetric phospholipid bilayer), the periplasm (peptidoglycan), and the outer membrane (phospholipid and lipopolysaccharide), is the target site of antibiotics, bacteriophages, and host immune systems; it is also the region of sensing and responding to pH, protein misfolding, and oxidative stress [134,135]. The major mechanisms of the bacterial envelope are the production of lipopolysaccharides (LPS) as well as capsule and outer membrane proteins [43]. Nevertheless, physiological functions such as permeability and efflux contribute to enhanced resistance to exogenous stresses. Bacterial envelope stress responses (ESRs) play an important role in the maintenance of membrane homeostasis, the sensing of environmental changes, and the repair of cellular damage [136]. The ESRs induce the dissociation of CpxP from the transmembrane sensor kinase (CpxA) that phosphorylates the cytoplasmic transcription (CpxR) and changes the expression of chaperons, foldases, and proteases; this influences antibiotic resistance [136,137]. The regulatory cascades of ESRs are induced by independent and overlapping stimuli, including the two-component signal transduction system (TCS) and RNA polymerase-associated alternative σ factor [136,137].

**Figure 9:**
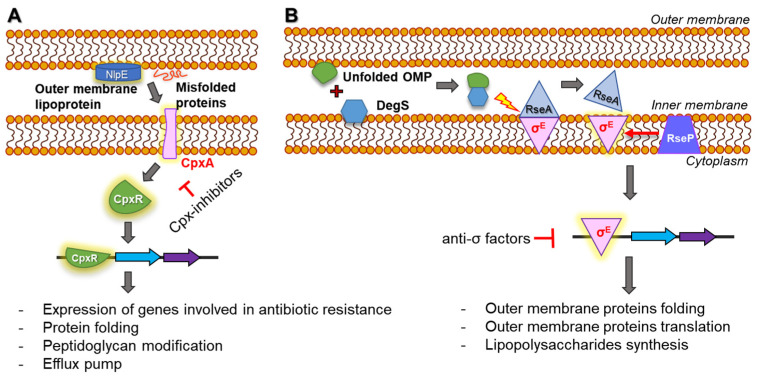
Cpx- (**A**) and σ^E^-regulated (**B**) envelope stress responses in bacteria [136].

There are Cpx- and σ^E^-regulated ESRs to inner membrane and periplasmic/outer membrane stresses, respectively [134,136]. The Cpx-TCS is activated by defects in protein secretion across the inner membrane and the misfolding of secreted cytoplasmic and periplasmic proteins (Figure 9A) [134]. The outer-membrane lipoprotein NlpE activates the Cpx-TCS in *E. coli* [138]. The factors that activate the Cpx-TCS include increased pH, altered osmotic pressure, cell adhesion to hydrophobic surfaces, and abnormal peptidoglycan production [139]. The maintenance of inner membrane homeostasis is associated with the transcriptional upregulation of peptidoglycan-modification-, efflux-, and redox-related genes triggered by the Cpx response [140]. The σ^E^ is activated by an aggregation of misfolded outer membrane proteins and lipopolysaccharides in the periplasm [141]. Therefore, the extracytoplasmic σ^E^ plays an important role in the maintenance of the bacterial outer membrane (Figure 9B) [142].

The ESR systems are responsible for the increase in antibiotic resistance that protects bacteria from cell-wall-synthesis-inhibiting and protein-synthesis-inhibiting antibiotics [143,144]. Additionally, the efflux pump systems that are activated by the ESR enhance the resistance to different classes of antibiotics, such as fluoroquinolones and aminoglycosides [145]. This suggests that the activation of Cpx is associated with the overexpression of efflux-pump-related genes, such as *tolC*, *mdtABC*, and *acrD*, leading to resistance to β-lactam, fluoroquinolone, and aminoglycosides [146]. RND efflux pumps such as AcrAB–TolC play a role in reducing antibiotic accumulation in the periplasmic space [122]. Additional mechanisms conferring antimicrobial peptide resistance in Gram-negative bacteria are related to a novel transcriptional regulatory pathway, the double-arginine translocation (Tat) system, which is regulated by the CpxR/CpxA system [147]. In addition, ESRs can reduce the accumulation of intracellular antibiotics by altering outer membrane permeability [148]. Furthermore, because the σ^E^ and its gene products are involved in vesicle formation, the outer membrane vesicles can transport antibiotics out of bacterial cells, resulting in enhanced antibiotic resistance [149,150]. Consequently, the σ^E^-regulated ESRs can protect bacterial cells from antibiotics by increasing the formation of outer membrane vesicles [151]. The σ^E^- and Cps-dependent ESRs are involved in the modulation of the cell membrane, conferring bacterial resistance to antibiotics. In the literature, the susceptibility of Cpx-deleted *S*. Typhimurium to β-lactams was increased due to the loss of envelope permeability [151]. Thus, ESRs can be considered potential targets in the effort to control antibiotic resistance. Transcriptional factor engineering has been proposed as a tool for regulating σ^E^ activity [152]. An inner membrane protein, RseA, negatively regulates σ^E^ as an anti-σ factor [153]. The *N*-terminal cytoplasmic domain of RseA inhibits σ^E^ activity, suggesting anti-σ factors can be used to design synthetic regulatory networks [154].

## 9. Osmotic-Stress-Induced Antibiotic Resistance

**Figure 10:**
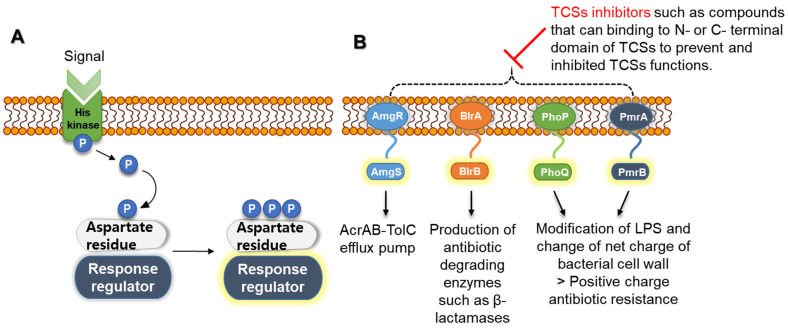
(**A**) Two-component signal (TCS) transduction pathways in bacteria and (**B**) TCSs related to antibiotic resistance [166].

Osmotic stress has significant effects on bacterial structure and physicochemical properties [155]. The cytoplasmic membrane acts as a permeability barrier, and it is involved in rapid solute fluxes through the transmembrane in response to osmolality changes [156]. Bacteria evolve mechanisms that underlie the changes in external osmotic stress [155]. Bacterial cells respond to osmotic stress at two different levels, including protein activity, which is an immediate response, and gene transcription, which is a long-term response [157]. The membrane-bound histidine kinases (TCSs) and membrane-bound chemoreceptors (chemotactic system) are common mechanisms responsible for the responses to bacterial osmotic stress [158,159]. The TCSs, consisting of membrane-bound sensor histidine kinases and soluble response regulator proteins, play an important role in bacterial survival and virulence in the cytoplasm [160]. The sensor histidine kinases detect specific signals from the periplasm, membrane, and cytoplasm. Subsequently, the signals are transferred to be auto-phosphorylated at a conserved histidine residue. The phosphate group is transferred to an aspartate residue in the soluble response regulators, leading to changes in gene expression (Figure 10A) [161].

TCSs may be associated with the development of antibiotic resistance [162]. TCS-induced antibiotic resistance can be explained by several processes, including the modification of cell surface components, a decrease in drug influx, an increase in drug efflux, the activation of antibiotic-degrading enzymes, and the formation of biofilms (Figure 10B) [162]. Positively charged antibiotics, such as aminoglycosides, colistin, and polymyxin B, exploit the negatively charged outer membrane of Gram-negative bacteria to induce membrane rupture and lead to bacterial cell death [163]. However, the TCSs, such as PhoPQ and PmrAB, are involved in the remodeling of bacterial surface components, leading to a decrease in antibiotic permeation due to the change in bacterial surface charge [164]. Furthermore, the colistin resistance in *E. coli* and *S.* Typhimurium is regulated by the PmrAB and PhoPQ regulatory systems [147]. In fact, colistin is able to bind to the phosphate group of LPS lipid A and then disrupt the outer membrane by removing the divalent cations Ca^2+^ and Mg^2+^, resulting in cell death [165]. However, the loss of these cations activates the TCSs PmrAB and PhoPQ, leading to the synthesis and transfer of cationic groups such as phosphoethanolamine (pEtN) and 4-amino-4-deoxy-L-arabinose (L-Ara4N). As a result, the LPS modifications reduce the negative charge on the outer membrane and hinder the binding site of colistin [165].

In the EnvZ/OmpR TCS, EnvZ, as a histidine kinase, detects osmolality changes and regulates the expression of the outer membrane porins (OmpC and OmpF), and OmpR, as a response regulator, activates the AcrAB–TolC MDR efflux pump and regulates the expression of OMPs [167,168]. The upregulation of transcriptional activators induces the activation of efflux pumps and the suppression of porin channels [169]. The repressor OmpX, triggered by environmental factors, negatively regulates the expression of OmpC, which is responsible for increased resistance to β-lactams and fluoroquinolones [6]. In addition, the AmgRS sensor kinase can prevent bacterial cell damage from aminoglycosides by upregulating the MexAB–OprM MDR efflux pump [170]. Another example of TCSs associated with antibiotic resistance is the production of antibiotic-hydrolyzing enzymes. The CreBC TCS activates the chromosomal *ampC*, encoding β-lactamase in *P. aeruginosa* [171]. The BlrAB TCS in *Aeromonas* also regulates the production of several β-lactamases, such as carbapenemase and penicillinase, through the phosphorylation of BlrA [172]. In addition, SagS and BfiSR regulate biofilm formation and promote antibiotic resistance [173]. Thus, the inhibition of TCSs can extend the spectrum of antibiotic activity [174]. The halogenated pyrrolo benzoxazines isolated from *Streptomyces rimosus* inhibit the auto-phosphorylation of histidine kinases and enhance antibacterial activity against *E. coli* [175]. In the literature, the inhibition of VanSR TCS by using cyanoacetoacetamide contributed to an increase in bacterial susceptibility to vancomycin [176].

## 10. Concluding Remarks

Adaptive stress responses are the survival strategies of bacteria when exposed to detrimental conditions and can mediate antibiotic resistance mechanisms. For example, the SOS response is induced by DNA damage that is repaired through recombination and mutagenesis, promoting horizontal gene transfer (HGT), which is responsible for the distribution of genes that increase virulence and antibiotic resistance. The heat shock response upregulates molecular chaperones and proteases for bacterial survival in the presence of antibiotics. The stringent response is mediated by (p)ppGpp and regulated by RelA and SpoT, which affect antibiotic susceptibility. Oxidative stress triggers the efflux pump systems that contribute to antimicrobial resistance. The cell envelope stress response is induced by antibiotics and regulated by alternate sigma factors and TCSs. It has been demonstrated that bacterial cells respond to osmotic stress through TCSs and membrane-bound chemoreceptors. In addition, there are several mechanisms induced by TCSs that relate to antibiotic resistance development. For example, TCSs are able to modify cell surface components, induce antibiotic efflux pump systems, produce antibiotic-degrading enzymes, and promote a bacterial antibiotic resistance lifestyle (biofilms). Consequently, the link between bacterial stress responses and the emergence of antibiotic-resistant bacteria shows that stress response regulators are involved in the development of resistance phenotypes. Therefore, it should be noted that stress response systems are associated with antibiotic resistance through a cascade of events. As a result, stress response pathways may be an appropriate target for therapeutic interventions. Therefore, it should be noted that the anti-regulation of bacterial stress responses can be a potential target in the effort to overcome the development of antibiotic resistance in bacteria. Further in-depth studies on the discovery of novel stress response inhibitors are essential to combat antibiotic resistance.

## Figures and Tables

**Table 1:** Virulence genes involved in stress-related sigma-factor-induced antimicrobial resistance in Gram-negative bacteria.

Sigma Factor	Virulence Genes	Bacteria Species	Virulence	Reference
σ^19^ (FecI)	*pvdS*, *hasI*	*Pseudomonas aeruginosa*	Activation of multidrug efflux pumps such as MaxAB–OprM	[22]
σ^24^ (RpoE)	*blaOXA*	*Klebsiella pneumoniae*	Reduction in the expression of porin protein and regulation of the transcription of the carbapenemase gene	[23]
	*araC*, *xylS*	*Salmonella* Typhi	Downregulation of outer membrane protein and activation of efflux pump system	[24]
	*bipA*	*Salmonella* Typhimurium	Resistance to antimicrobial peptides	[25]
σ^28^ (RpoF)	*flbB*, *flaI,*	*Escherichia coli*	Flagella synthesis	[26]
	*fliA*	*Dickeya dadantii*, *Vibrio cholera*	Flagella synthesis	[27,28]
σ^32^ (RpoH)	*mtrE*	*Neisseria gonorrhoeae*	Adhesion to host cells and activation of multidrug efflux pump	[29]
σ^38^ (RpoS)	*acrA*, *acrB*, *tolC*	*Salmonella* Typhimurium	Regulation of the transcription of multidrug-efflux-pump-related genes	[30]
	*adhE*	*Escherichia coli*	Biofilm formation	[31]
	*bolA*	*Escherichia coli*	Biofilm formation and regulation of the transcription of the β-lactamase gene (*ampC*)	[26]
	*bpsl*	*Burkholderia pseudomallei*	Acyl-homoserine lactone synthesis and quorum sensing	[32]
	*flhDC*	*Yersinia pseudotuberculosis*	Flagella synthesis	[33]
	*grxB*	*Cronobacter sakazakii*	Acid tolerance, auto-aggregation, and biofilm formation	[34]
	*mutS*	*Salmonella Typhimurium*, *Pseudomonas aeruginosa*	Antibiotic resistance by increasing mutation frequency and generating adaptive mutations	[35]
	*ndvB*	*Pseudomonas aeruginosa*	Biofilm formation	[36]
	*sefA*	*Salmonella* Enteritidis	Fimbrial protein synthesis	[37]
	*spv*	*Salmonella* Typhimurium	Increase in cytotoxic effect on host cells and requirement for delayed cell death by apoptosis	[38]
	*ycfR*	*Salmonella* Typhimurium, *Salmonella saintpaul*	Induction of bacteria adhering to the surface	[39]
σ^54^ (RpoN)	*dbpA*	*Borrelia burgdorferi*	Regulation of biofilm formation by facilitating the adherence of bacteria to the extracellular matrix	[40]
	*hcp1*	*Vibrio alginolyticus*	Regulation of the expression of quorum-sensing-related genes and biofilm formation	[41]
	*hrp*	*Erwinia amylovora*	Flagellar motility and pilus-mediated attachment	[42]
	*lacZ*	*Pseudomonas aeruginosa*	Quorum sensing	[43]
	*qrr*	*Vibrio parahaemolyticus*	Regulation of quorum sensing, the production of capsule polysaccharides, and bacterial motility	[44]
σ^70^ (RpoD)	*ttgA*, *ttgB*, *ttgC*	*Pseudomonas putida*	Activation of multidrug efflux pumps such as TtgABC	[45]

## Data Availability

Not applicable.
